# Psychometric properties of the short Warwick Edinburgh mental well-being scale (SWEMWBS) in service users with schizophrenia, depression and anxiety spectrum disorders

**DOI:** 10.1186/s12955-017-0728-3

**Published:** 2017-08-01

**Authors:** Janhavi Ajit Vaingankar, Edimansyah Abdin, Siow Ann Chong, Rajeswari Sambasivam, Esmond Seow, Anitha Jeyagurunathan, Louisa Picco, Sarah Stewart-Brown, Mythily Subramaniam

**Affiliations:** 1 Research Division, Institute of Mental Health, 10 Buangkok View, Singapore, 539747 Singapore; 2 Warwick Medical School, University of Warwick, Coventry, London, UK

**Keywords:** Confirmatory factor analysis, Convergent and divergent validity, Mental health, Asian

## Abstract

**Background:**

To establish the validity and reliability of the Short Warwick Edinburgh Mental Well-being Scale (SWEMWBS) in service users with schizophrenia, depression and anxiety spectrum disorders in Singapore and estimate SWEMWBS scores across socio-demographic and the three psychiatric diagnostic groups in the sample.

**Methods:**

This secondary analysis was conducted using data from a study among outpatients of a tertiary psychiatric hospital. In addition to the SWEMWBS, socio-demographic data and current psychiatric diagnosis were collected. Service users were also administered the Global Assessment of Functioning (GAF), Patient Health Questionnaire (PHQ)-8, Generalised Anxiety Disorder (GAD)-7, Satisfaction with Life Scale (SWLS) and the Positive Mental Health (PMH) instrument. The SWEMWBS was tested for factorial validity, reliability and convergent and divergent validity.

**Results:**

In total, 350 service users with a mean (SD) age of 39.1 (11.1) years were included in this study of which 39.4%, 38.9% and 21.7% had schizophrenia, depression and anxiety spectrum disorders, respectively. The single factor structure of the SWEMWBS was confirmed by confirmatory factor analysis (CFI = 0.969, TLI = 0.954, RMSEA = 0.029). The internal consistency reliability was high (Cronbach’s alpha = 0.89). The convergent and divergent validity testing revealed that the SWEMWBS scores had significant moderate to high positive correlations with GAF, SWLS and PMH scores and moderate negative correlations with (PHQ)-8 and (GAD)-7 scores. SWEMWBS scores were higher in married participants (22.2 (5.4) versus never married: 20.7 (5.3) and divorced/separated/widowed: 20.4 (5.1), *p* = 0.049) and among those with schizophrenia (22.8 (5.5) versus depression:19.6 (4.7) and anxiety spectrum disorders 20.9 (5.2), *p* < 0.001).

**Conclusion:**

These results demonstrate adequate validity and reliability of the SWEMWBS in people with schizophrenia, depression and anxiety spectrum disorders in Singapore.

## Background

An estimated one fourth of the world population has a mental illness, with mental and substance use disorders being the leading cause of years lost due to disability [1, 2]. Although reducing the severity of symptoms is an important goal in the treatment of mental disorders, it is proposed that merely reducing symptoms does not indicate mental health and well-being in this population or reduce their disability [3]. It is well established that apart from psychological and emotional well-being, mental illnesses have a significant negative impact on the functioning and social well-being of those affected [4].

In 2004, the World Health Organisation (WHO) defined mental health as “a state of complete physical, mental and social well-being and not merely the absence of disease” [5]. This was a major advancement, particularly for those affected with and at risk of developing mental disorders as this distinction between mental illness and mental health supported the positive social and behavioral model for mental health first proposed by Jahoda over a negative psychiatric symptoms approach [6]. Over the past decade, there has been a considerable impetus on improving mental health and well-being in different populations and creating health-oriented rather than illness-oriented interventions, for people with mental illnesses [3, 7, 8]. There has consequently been a greater focus on intervening on all aspects of mental well-being in those affected with mental illnesses and complementing psychiatric treatment with interventions such as mindfulness that are geared towards strengthening mental health [9, 10].

From the evidence and initiatives across the globe, mental wellness-based interventions are playing a crucial role in public and mental health research and practice [3, 7–9]. Measuring mental health and well-being in individuals provides unique information concerning emotional and social well-being of a person which may not be available by using psychiatric assessments alone. However, there is as yet no consensus on what constitutes mental well-being [11]. Mental well-being literature posits two dominant theories – hedonic or subjective well-being which relates to subjective appraisal of happiness and life satisfaction and eudaimonic or psychological well-being comprising individuals’ psychological functioning and self-actualization [11]. With the lack of one universally accepted definition of well-being, the existing well-being measures have adopted different frameworks of well-being for their development. While scales such as the Positive and Negative Affect Schedule (PANAS) and Satisfaction With Life Scale (SWLS) relate to hedonic aspects of well-being the Affectometer relates to individual 's functioning and feelings, and the Ryff Scales of Psychological Well-being measure aspects of well-being representing self-acceptance, establishment of quality ties to others, sense of autonomy in thought and action, ability to manage complex environments to suit personal needs and values, pursuit of meaningful goals and a sense of purpose in life, and continued growth and development as a person [12–14]. On the other hand the WHO-five well-being scale provides a measure of overall well-being including physical and mental health aspects [15]. There is therefore a dearth of measures that can comprehensively assess the multiple components of mental well-being.

The Warwick Edinburgh Mental Well-being Scale (WEMWBS) was developed in the United Kingdom to “capture a wide conception of well-being, including affective-emotional aspects, cognitive-evaluative dimensions and psychological functioning” using a short tool that can be used as monitoring tool for positive aspects of mental health in population studies [16]. This unidimensional scale comprises 14 positively worded items representing both hedonic and eudaimonic aspects of mental health including feelings of optimism, cheerfulness and relaxation), satisfying relationships and positive energy, clear thinking, self-acceptance, personal development, competence and autonomy. Since its initial development, the scale has been used to assess mental well-being in a number of epidemiological and interventional studies [17–19]. Subsequently, a shorter version of the measure the Short Warwick Edinburgh Mental Well-being Scale (SWEMWBS) was developed [20]. This tool has 7 items representing a unidimensional well-being structure, similar to its original longer version however the short version tends more towards psychological and eudemonic well-being, with fewer items covering hedonic well-being or affect. The SWEMWBS was however found to be psychometrically more robust than the longer version and with the advantage of its additional brevity, has been used in a number of population studies globally. It has been validated among the Scottish adult population and veterinary professionals [20, 21]. It has also been translated in Chinese, Norwegian and Swedish languages and validated in respective populations [22, 23] as well as used in the evaluation of well-being interventions [24, 25]. The scale has also been employed to measure well-being in other populations such as older adults, stigmatised minorities and patients with schizophrenia [26–28]. Although Mezquida & Fernandez-Egea [28] and Ng et al. [22] have used the scale in service users with mental disorders, psychometric properties of the English language version of the scale have not been investigated in this population.

An accurate and appropriate assessment of mental well-being is critical in determining the effectiveness of clinical and psychosocial interventions as well as for the formulation of policies for people with mental illnesses. The extent to which mental well-being measures are valid for people with mental disorders has however been insufficiently examined. A recent study in Singapore assessed the level of positive mental health (PMH) among people with schizophrenia, depression and anxiety spectrum disorders and validated the PMH instrument in this population [29].

The PMH instrument covers six domains of PMH: general coping, emotional support, spirituality, interpersonal skills, personal growth and autonomy and global affect representing psychological and subjective well-being largely similar to the SWEMBS except for the additional component of spirituality. The PMH instrument however has 47 items that make employing it for assessments in routine practice challenging. Given the similarity in the conceptualization of the two tools, it was of interest to find out the psychometric properties of the shorter tool. Using data from a study among service users with mental disorders in Singapore, the current work aimed to establish the validity and reliability of the SWEMWBS in adults with schizophrenia, depression and anxiety spectrum disorders. The study also aimed to estimate SWEMWBS scores across socio-demographic and psychiatric diagnostic groups in this sample.

## Methods

### Survey and sample population

Ethics approval was obtained for the study from the Institutional Ethics Committee, the Domain Specific Review Board of the National Healthcare Group, Singapore. The survey was a cross-sectional study conducted on a convenient sample of adult service users seeking treatment at the outpatient clinics of Institute of Mental Health, the only tertiary psychiatric hospital in Singapore. Data from 350 service users aged 21 to 65 years with a history of International Classification of Disease version 9 schizophrenia, depression or anxiety spectrum disorders, who were Singapore citizens and Permanent Residents of Chinese, Malay or Indian ethnicity was used for this analysis. All participants provided informed consent and were literate in English. They were enrolled into the study using a quota sampling plan to obtain adequate representation by diagnosis, age, gender and ethnicity. Participants were self-referred or clinician-referred. Posters informing service users of the ongoing study and its eligibility criteria were placed in the clinics along with the phone numbers and email addresses of the study team members. Psychiatrists and other healthcare professionals were also requested to refer their clients for the study. Further details on the study recruitment process and participants are published elsewhere [29].

### Measures

Socio-demographic and clinical information on the participants including their age, gender (men/ women), ethnicity (Chinese, Malay or Indian), educational level (some/primary, secondary, vocational, A level, diploma or tertiary), marital status (never married, married or divorced/ separated/ widowed), employment status (unemployed, employed, student, retired or homemaker) and psychiatric diagnosis (schizophrenia, depression or anxiety) was collected upon enrolment in the study through self-report and review of their administrative records.

The SWEMWBS was used to measure mental well-being by asking participants how often they had been ‘feeling optimistic about the future; feeling useful; feeling relaxed; dealing with problems well; thinking clearly; feeling close to other people; able to make up their own mind about things’ over the past 2 weeks [20]. Responses ranged from 1 (none of the time) to 5 (all of the time) on a 5-point Likert scale. Raw item-scores were summed and converted to metric total score using SWEMWBS conversion table [20]. SWEMWBS score ranged from 7 (lowest possible mental well-being) to 35 (highest possible mental well-being).

In this study, PMH was assessed with the PMH instrument which is a validated self-report measure developed in Singapore [30, 31]. Participants were presented with the statements representing the six PMH domains, such as ‘When I feel stressed I try to solve the problem one step at a time’, ‘I get along well with others’ and ‘I feel comfortable expressing my opinions’, and asked to select a number showing how much the item described them on a scale from 1 to 6 (1- ‘Not at all like me’ to 6- ‘Exactly like me’). Total PMH score was obtained by adding item scores and dividing by 47 (the number of items in the scale) to give a score ranging from 1(low PMH) to 6 (high PMH).

The Global Assessment of Functioning (GAF) scale [32] was administered by trained research team members to assess the psychological, social and occupational functioning of the participants. Using uniform semi-structured interviewing, the level of functioning was rated on a scale of 0 to 100 where higher scores indicated better functioning.

Participants were also asked to complete the 5-item SWLS indicating satisfaction with their lives on a 7-point scale from ‘strongly disagree’ to ‘strongly agree’ where higher total score indicated higher life satisfaction [12].

Depressive symptoms and severity were measured using the Patient Health Questionnaire (PHQ)-8 [33]. Participants were asked eight statements on how often in the past 2 weeks, they had been bothered by problems associated with depression such as ‘feeling down or depressed or hopeless’ and having ‘trouble concentrating on things’ on a 4-point scale where 0 = not at all and 3 = nearly every day. Similarly, the Generalised Anxiety Disorder (GAD)-7 Scale included 7 items that measured anxiety symptoms such as ‘feeling nervous, anxious or on edge’ and having ‘trouble relaxing’ [34]. Participants were asked to indicate how often they had been bothered by these problems in the past 2 weeks on a 4-point scale (‘not at all’ to ‘nearly every day’). For both these scales, total item scores were obtained with higher scores indicating greater depression and anxiety symptoms and/or severity.

### Statistical analyses

The factorial validity of the measure was assessed using confirmatory factor analysis (CFA) on the overall sample and across the three diagnostic groups. MPLUS software was used to conduct the CFA using polychoric item correlations matrix with weighted least squares with mean-adjusted chi-square statistic estimator. All items were treated as categorical variables. Three goodness-of-fit (GOF) indices were estimated: comparative fit index (CFI), Tucker-Lewis index (TLI) and root mean square error of approximation (RMSEA) with cut-off values of more than 0.95 for TLI and CFI and less than 0.06 for RMSEA [35]. Other statistical analyses were performed using SPSS, version 18. Descriptive statistics were derived for continuous and categorical variables. Reliability of the SWEMWBS was assessed by Cronbach’s alpha coefficient for internal consistency. Convergent and divergent validity of the SWEMWBS was determined with Pearson’s coefficients for correlations between SWEMWBS and convergent and divergent measures to test apriori hypothesis (Table 1). Correlations of the SWEMWBS score with total PMH, SWLS and GAF scores assessed its convergent validity while correlations with (PHQ)-8 and (GAD)-7 scores tested divergent validity. Pearson’s correlation was used to assess the relation of age with SWEMWBS and differences in the SWEMWBS scores across the different socio-demographic and diagnostic groups were estimated using independent t-tests or ANOVA with Bonferroni post-hoc tests. Linear regression analysis was used to explore the relationship between socio-demographic variables and diagnosis in relation to the SWEMWBS score. The level of significance for all statistical tests was set at 0.05.

**Table 1:** Convergent and Divergent validity measures and hypotheses tested in the study

Measures	Description	Hypothesis / validity criteria^a^
Positive Mental Health (PMH) instrument	This 47-item multi-dimensional scale measures positive mental health, where scores range from 1 to 6, with higher scores indicating higher positive mental wellbeing. The response scale represents 1- ‘Not at all like me’ to 6- ‘Exactly like me’	SWEMWBS will have moderate to high positive correlation with PMH total score.
Global assessment of functioning (GAF)	The Global Assessment of Functioning scale is a scoring system for the severity of illness in psychiatry which assesses overall functioning, taking into account impairments in psychological, social and occupational/school functioning. The scale ranges from 0 (inadequate information) to 100 (superior functioning).	SWEMWBS will have moderate positive correlation with GAF score.
Satisfaction with Life Scale (SWLS)	This 5-item scale measures global cognitive judgments of satisfaction with one’s life, using a 7-point scale from 1 = strongly disagree to 7 = strongly agree. Scores are summed and higher scores indicate higher satisfaction.	SWEMWBS will have mild to moderate positive correlation with SWLS.
Patient Health Questionnaire (PHQ) -8	This is a self-administered depression scale that adopts a 4-point scale, where 0 = not at all and 3 = nearly everyday and respondents indicate how often they have been bothered by each of the items, in the past 2 weeks. Total scores range from 0 to 27, where scores of 20 and above indicate severe major depressive disorder.	The SWEMWBS will have mild to moderate negative correlation with PHQ-8.
General Anxiety Disorder (GAD) -7 scale	In this 7-item anxiety measure respondents are asked in the past 2 weeks how often they have been bothered by the following problems and use a 4-point scale from 0 being ‘not at all sure’ to 3 representing ‘nearly every day’. Scores are summed and higher scores indicate greater anxiety.	The SWEMWBS will have mild to moderate negative correlation with GAD-7.

^a^Strength of correlations: mild: *r* = 0.2–0.4; moderate: *r* = 0.5–0.7; high: *r* = 0.8–1.0

## Results

The study included a sample of 350 respondents aged 21–65 years with a mean age (SD) of 39.1 (11.1) years. There were almost equal proportion of men and women, and the majority were of Chinese ethnicity (40.9%), never married (56.0%) and with secondary level education (34.3%) (Table 2). Of the participants, 39.4% had schizophrenia, 38.9% had depression and 21.7% had anxiety spectrum disorders.

**Table 2:** Socio-demographic characteristics of the study sample and SWEMWBS sub-group scores (*N* = 350)

				SWEMWBS score		
		n	%	Mean	SD	*P* value
Age (Mean, SD)	39.1, 11.1					
Gender	Men	178	50.9	20.9	5.3	0.346
	Women	172	49.1	21.4	5.4	
Ethnicity	Chinese	143	40.9	20.8	4.6	0.560
	Malay	102	29.1	21.1	5.7	
	Indian	105	30.0	21.6	5.9	
Marital status	Never married	196	56.0	20.7	5.3	0.042^#^
	Married	105	30.0	22.2	5.4	
	Divorced/ Separated/ Widowed	49	14.0	20.4	5.1	
Education level	Some/Primary	34	9.7	20.9	6.4	0.637
	Secondary	120	34.3	20.6	4.8	
	Vocational	39	11.1	21.7	5.5	
	A Level	27	7.7	22.3	5.7	
	Diploma	86	24.6	21.4	5.8	
	Tertiary	44	12.6	21.1	4.6	
Employment status	Unemployed	158	45.3	20.8	5.6	0.495
	Employed	162	46.4	21.4	5.3	
	Student	12	3.4	22.0	4.0	
	Retired	7	2.0	22.9	4.4	
	Homemaker	10	2.9	19.4	1.6	
Diagnostic group	Schizophrenia	138	39.4	22.8	5.5	<.001^$^
	Depression	136	38.9	19.6	4.7	
	Anxiety	76	21.7	20.9	5.2	

^#^Statistical difference in SWEMWBS score was not observed by marital status in Bonferroni post-hoc test

^$^Participants with schizophrenia have higher SWEMWBS score than those with depression (*p* < 0.001), and anxiety spectrum disorders (*p* = 0.034) using Bonferroni post-hoc test

The unidimensional structure of the SWEMWBS was confirmed via CFA (Table 3). The GOF indices were satisfactory (CFI = 0.969, TLI = 0.954, RMSEA = 0.029). The structure of SWEMWBS was strong among patients with schizophrenia with almost all GOF indices meeting the set criteria (CFI = 0.967, TLI = 0.950, RMSEA = 0.059). SWEMWBS met two of the three GOF indices among those with depression and all three indices among those with anxiety spectrum disorders (Table 4).

**Table 3:** Confirmatory factor analysis for structure of SWEMWBS among people with schizophrenia, depression and anxiety spectrum disorders

Items	Mean	SD	Kurtosis	Skewness	Item-rest correlation	Factor loading
Feeling optimistic about the future	3.16	1.202	−0.805	−0.067	0.673	0.709
Feeling useful	3.22	1.182	−0.767	−0.135	0.742	0.784
Feeling relaxed	3.13	1.085	−0.530	−0.039	0.708	0.758
Dealing with problems well	3.14	1.102	−0.630	−0.112	0.731	0.793
Thinking clearly	3.36	1.116	−0.609	−0.273	0.774	0.832
Feeling close to other people	3.09	1.203	−0.862	−0.127	0.640	0.668
Able to make up my own mind about things	3.42	1.085	−0.612	−0.212	0.650	0.688
Reliability indices						
Cronbach’s alpha						0.898
Congeneric						0.903
Fit indices						
Chi-square test						30.47
Degree of freedom						14
CFI						0.969
TLI						0.954
RMSEA						0.029

**Table 4:** Confirmatory factor analysis for structure of SWEMWBS among people with schizophrenia, depression and anxiety spectrum disorders separately

Items	Schizophrenia	Depression	Anxiety
	Factor loading		
Feeling optimistic about the future	0.683	0.719	0.685
Feeling useful	0.696	0.771	0.877
Feeling relaxed	0.766	0.704	0.764
Dealing with problems well	0.713	0.822	0.819
Thinking clearly	0.777	0.845	0.815
Feeling close to other people	0.625	0.751	0.607
Able to make up my own mind about things	0.626	0.694	0.798
Fit indices			
Chi-square test	20.716	25.785	12.01
Degree of freedom	14	14	14
CFI	0.967	0.966	1.000
TLI	0.950	0.949	1.000
RMSEA	0.059	0.079	<0.01

Cronbach’s alpha coefficient for the overall sample was 0.90 while that among the three patient groups was 0.87 for schizophrenia, 0.90 for depression and 0.91 for anxiety, indicating high internal consistency reliability.

SWEMWBS scores significantly and positively correlated with the convergent validity measures, with significant moderate to high correlation with the SWLS and the total PMH scores (Table 5). However the convergence with GAF was only partially fulfilled as the correlation coefficient although significant and in the positive direction, was lower than 0.5. As expected the SWMWBS showed significant, negative and moderate correlations with the (PHQ)-8 and (GAD)-7 scores.

**Table 5:** Correlation coefficients for SWEMWBS with validity measures

	SWEMWBS	
	Pearson’s correlation coefficient, r	*P* value
Convergent measures		
*PMH*	0.779	<.001
*GAF*	0.437	<.001
*SWLS*	0.664	<.001
Divergent measures		
*PHQ-8*	−0.504	<.001
*GAD-7*	−0.517	<.001

Abbreviations: *PMH* Positive Mental Health, *GAF* Global Assessment of Functioning, *SWLS* Satisfaction with Life Scale, *PHQ-8* Patient Health Questionnaire, *GAD-7* Generalised Anxiety Disorder Scale

The mean (SD) SWEMWBS score in the sample was 21.1 (5.3) and ranged from 7 to 35. Table 2 displays the SWEMWBS scores across socio-demographic and diagnostic groups, where scores differed significantly by age groups, marital status and psychiatric diagnosis. SWEMWBS scores were positively and significantly correlated with age (Pearson’s Correlation coefficient: 0.19, *p* < 0.001). SWEMWBS mean (SD) scores were significantly higher among those who were married (22.2 (5.4) versus never married: 20.7 (5.3) and divorced/separated/widowed: 20.4 (5.1), *p* = 0.049) and among those with schizophrenia (22.8 (5.5)) versus those with depression (19.6 (4.7)) and anxiety spectrum disorders (20.9 (5.2), *p* < 0.001).

After adjusting for socio-demographic variables and psychiatric diagnosis in the regression analysis for SWEMWBS score, age was found to be positively associated (β (SE):0.10 (0.03), *p* < 0.001) with mental well-being along with Indian ethnicity (β (SE):1.55 (0.66), *p* = 0.02, versus Chinese) (Table 6). On the other hand, being never married (versus married), having secondary level education (versus tertiary), being unemployed (versus employed) and having depression or anxiety (versus schizophrenia) were associated with lower SWEMWBS score.

**Table 6:** Socio-demographic correlates of SWEMWBS among people with schizophrenia, depression and anxiety spectrum disorders

		β	SE	95% CI		*P* value
				Lower	Upper	
Age		0.10	0.03	0.04	0.15	<.001
Gender	Women	Ref				
	Men	−0.42	0.54	−1.47	0.63	0.43
Ethnicity	Chinese	Ref				
	Malay	1.04	0.68	−0.30	2.38	0.13
	Indian	1.55	0.66	0.25	2.85	0.02
Marital status	Married	Ref				
	Never married	−1.45	0.69	−2.81	−0.10	0.04
	Divorced/ Separated/ Widowed	−0.99	0.88	−2.72	0.74	0.26
Education level	Tertiary	Ref				
	Some/Primary	−1.14	1.20	−3.49	1.21	0.34
	Secondary	−1.67	0.61	−3.46	−0.11	0.04
	Vocational	0.91	1.08	−1.21	3.04	0.40
	A Level	0.97	1.20	−1.39	3.32	0.42
	Diploma	0.66	0.91	−1.14	2.45	0.47
Employment status	Employed	Ref				
	Unemployed	−1.27	0.58	−2.40	−0.14	0.03
	Other (Student/Retired/Homemaker)	−0.17	1.02	−2.16	1.83	0.87
Diagnostic group	Schizophrenia	Ref				
	Depression	−3.78	0.63	−5.02	−2.54	<.001
	Anxiety	−2.50	0.78	−4.03	−0.97	<.001

## Discussion

Well-being measures have proven valuable in assessing the state of mental health in different populations, especially among the general population [17, 18, 30]. In recent years, well-being measures have been administered to youth, adults and elderly with mental conditions to understand the psychological and social reserves in people affected with mental illnesses [22, 28, 36–38]. Considering the social, cultural and economic variations in individuals, the testing of psychometric properties of well-being measures in different populations is an important step to ensuring the quality of assessment and future cross-cultural adaptation of well-being measures [39, 40].

The WEMWBS and SWEMWBS have been used routinely in national surveys and research investigations across United Kingdom, Europe and Australia and have generated extensive information on mental well-being and effects of well-being interventions in these populations [17–19, 22–28]. The present study, the first validation study on SWEMWBS conducted in a multi-ethnic group of service users with psychiatric conditions, determined the reliability and validity of the SWEMWBS in a multi-ethnic sample of mental health service users in Singapore. Our results provide evidence that the SWEMWBS is a valid and reliable measure for assessing mental well-being in patients with schizophrenia, depression and anxiety spectrum disorders.

With regard to factorial validity, the CFA showed that the factor structure identified among services users with mental disorders aged 21–65 years was consistent with that identified of the SWEMWBS in the Scottish population survey in adults aged 16 to 74 years [20] and Swedish and Norwegian general managers and managers [23]. The unidimensionality of the measure was supported by satisfactory fit indices (Table 3). Internal consistency was calculated using Cronbach’s alpha coefficient and demonstrated adequate homogeneity (α ≥ 0.70) in the study. As in its original validation in the Swedish adult population (α = 0.845) [20], Chinese version in the Hong Kong psychiatric service users (α = 0.89) [22], Norwegian and Swedish samples (α = 0.84 & α = 0.86 respectively) [23], the SWEMWBS performed reliably in the overall multi-ethnic Asian mental health service users in Singapore.

Convergent and divergent validity, which was explored with measures of positive and negative mental health, confirmed our assumptions. SWEMWBS scores were negatively correlated with the severity of depression and anxiety symptoms (PHQ-8 and GAD-7 scores) (Table 4). However, these correlations were moderate in strength, stressing the particular interest in relying on well-being measurements to complement clinical approaches, which do not encompass the entire experience of people with mental illnesses. As expected, individuals who had better PMH also had higher mental well-being. The relation between PMH and SWEMWBS scores showed the strongest positive correlation which was expected given that both the measures represent aspects of psychological and subjective well-being [20, 30]. This finding indicates that the two tools measure the same construct of mental well-being. In an earlier work with the SWEMWBS in a general population sample in Singapore [30], a moderate correlation of 0.660 between the PMH and the SWEMWBS scores was observed, which was lower than the current estimate. It is possible that the comparative homogeneity in emotional experiences in service users improved the congruence between these measures.

SWEMWBS also showed moderate correlation with SWLS scores as seen during its initial development [20] showing that the measure covers the subjective well-being aspect of satisfaction with life. Finally, the positive relationships between mental well-being and global functioning confirmed that service users’ functioning was in part related to their mental well-being. The correlations were moderate as expected because the GAF scores were a combination of both psychiatric symptoms and occupation and social well-being [32]. In addition, subjective measures such as quality of life and functioning have often showed only moderate correlation between self-assessment by individuals and their assessment by clinicians and raters [41]. A possible reason being that assessment of functioning concerning psychiatric symptoms and aspects of participants’ daily life such as work and social life by clinicians and raters often lays greater emphasis on negative experiences that may be relatively less important to the individuals. This may explain the weaker correlation between self-rated SWEMWBS and rater-assessed GAF scores in our study. Similar results were obtained for the correlation with PMH instrument and GAF scores in an earlier study [29]. Although of moderate strength, the positive correlation was nevertheless as expected, fulfilling the convergent validity criterion.

The study also estimated mental well-being using SWEMWBS score among service users with schizophrenia, depression and anxiety spectrum disorders in Singapore and found that the mean (SD) score of 21.1 (5.3) was slightly lower than the estimated 23.7 (SD = 3.78) among veterinary professionals in United Kingdom [21]. Although this was expected as low mental well-being has been previously reported in patient populations in comparison to the general population in Singapore [37], the difference of 0.5 SD in the two values was not high. It is possible that given that the patients in this sample were stable outpatients living in the community, they had access to psychosocial resources such as greater social networks and interactions than those having severe symptoms or restrictions faced by institutionalised patients [42], thereby reflecting mental well-being values closer to general populations. The higher mean of SWEMWBS in patients also suggests that mental well-being is a viable goal for people with mental disorders. Future research needs to focus on developing and studying interventions that can improve mental well-being in patients with mental illnesses that can lead to improved health and social outcomes in this group.

The study also identified factors associated with SWEMWBS. Contrary to the findings by Ng et al. [22], who did not find any association between the age of service users in Hong Kong and SWEMWBS score, in this study age was positively correlated with SWEMWBS scores. While studies with the WEMWBS and SWEMWBS elsewhere have also generated inconsistent results for age, older age was observed to be associated with higher mental well-being in a study with the multidimensional PMH instrument in the Singapore general population [43] and in people with mental disorders [36, 37]. The higher overall PMH score in these studies was attributed to the higher spirituality score in the older population in Singapore [43]. However given the unidimensional structure of SWEMWBS, further research is needed to understand the association of age with mental well-being as assessed with this measure. In terms of education, while well-being scores were correlated to the number of years of education in Hong Kong, in our sample scores differed between only those with secondary level education compared to tertiary education after adjusting for confounders (Table 6). Similar findings were observed for employment versus the unemployed. Research on the influence of higher education and employment on a person’s well-being is not conclusive. While it is proposed that these factors improve psychological and subjective well-being by improving learning, self-esteem and socio-economic condition of a person [44], others have attributed reported greater inclination towards material means, complex work tasks and other pressures to relate to low level of subjective well-being and life satisfaction [45]. We, however, did not collect such data in the current cross-sectional study could have provided greater understanding of the influence of these factors on mental well-being.

We also found that those with schizophrenia had highest SWEMWBS score which was similar to our observation with the PMH total scores in service users [37]. To our knowledge, differences in the level of mental well-being as assessed with the SWEMWBS or PMH instrument have not been investigated across psychiatric disorders in the past. Studies that have investigated well-being in people with schizophrenia have attributed their level of general mental well-being to the severity of their psychotic symptoms, years with illness and their health behaviours [22, 46]. However, past research on appraisals of subjective and psychological well-being have reported lower well-being levels in affective disorders such as depression and anxiety compared to schizophrenia or paranoid disorders. One explanation is the influence of cognitive process variation in schizophrenia and affective disorders like depression. Valiente et al. (2012) reported difference in metacognitive beliefs in paranoia and depression that relate to their perception of psychological well-being where “individuals with paranoia use self-consciousness (defined as the process of directing attention towards the self) to monitor self-threats” [47, 48]. The study found low psychological well-being in the domains of self-acceptance and autonomy in depression versus those with persecution delusions and healthy controls. It is proposed that having a higher sense of well-being could serve as positive sense of self in paranoia [49, 50], whereas the low self-consciousness seen in depression can exert an adverse effect [51]. Another theory is that of association of perceived stigma with life satisfaction or subjective well-being. Markowitz (1998) found stigma had higher association with life satisfaction in patients with depressive-anxiety symptoms that psychotic symptoms and attributed it to the lower self-esteem and discouragement from discrimination in affective disorders [52]. However, a third explanation to the difference in the mental well-being in type of mental illness could be the difference in self-report and recall process in schizophrenia compared to affective disorders. While current rating of subjective well-being in schizophrenia might be due to emotional withdrawal, blunt affect or poor insight, in affective disorders, it could be due to biased cognition insight, thus highlighting the challenges in comparison of self-report measures in patients with psychiatric problems [53].

Given the level of mental well-being was lower among patients with depression and anxiety also raises the question of whether well-being measures provide mere opposing assessment of psychiatric symptoms in affective conditions. The two continua model proposed by Keyes discusses the relationship between mental illness and mental health and urges the need to consider mental health as continuum over a dichotomy [54]. While highlighting that both mental health and mental illnesses lack specific diagnostic tests and are identifiable from a collections of symptoms of the underlying state, the theory proposes categorising people with mental illness into two groups of mental health - those who are “flourishing” defined as exhibiting high levels on at least one measure of hedonic well-being (happiness, life satisfaction, etc) and at least six measures of psychological well-being or positive functioning (autonomy, purpose in life, environmental mastery, etc.) and those “languishing” defined as not exhibiting these minimum requirements of flourishing. Given that the SWEMWBS is a unidimensional measure comprising both subjective/hedonic and psychological well-being, possess challenges in assessing how patients with depression fare in terms of these criteria. Further research addressing the concept of the two continuum model will be necessary to investigate the difference in the well-being score of depression or affective disorders versus schizophrenia.

Nevertheless, the study provides preliminary information on the association of the SWEMWBS score with important socio-demographic factors and diagnostic groups in adult mental health service users which can be useful while planning interventional studies in these populations. It is, however, important to further assess mental well-being determinants across these diagnostic groups after controlling for the effect of additional clinical, social and functional confounders. Moreover, due to the small sample size, it was not possible to assess measurement invariance in the SWEMWBS scores between the three diagnostic groups and the study was under-powered for conducting detailed analysis. Hence, further studies in larger samples are needed to assess these relationships.

This study contributes to the limited evidence on the psychometric properties of mental well-being measures in mental health service users and is of particular importance given the increasing application of the SWEMWBS in the evaluation of mental health oriented interventions in this population. However, certain limitations of the study need to be considered. Restricting the study inclusion to English-literate participants and those who were stable enough to provide consent and self-administer the questionnaires, convenient sampling of service users belonging to only three types of mental disorders and inability to collect attrition information on non-respondents are important limitations of the study and should be addressed in future studies.

## Conclusion

These results support the validity and reliability of the SWEMWBS in evaluating mental well-being in people with schizophrenia, depression and anxiety spectrum disorders. These data confirm that the SWEMWBS can provide a quick means of assessing/monitoring mental well-being in a population prone to poor mental health. Further research on its test re-test reliability and application in interventional studies is needed in Singapore.

## Abbreviations

CFA: Confirmatory factor analysis
CFI: Comparative fit index
GAD: Generalised anxiety disorder
GAF: Global Assessment of functioning
GOF: Goodness-of-fit
PHQ: Patient health questionnaire
PMH: Positive mental health
RMSEA: Root mean square error of approximation
SWEMWBS: Short warwick edinburgh mental well-being scale
SWLS: Satisfaction with life scale
TLI: Tucker-lewis index
WEMWBS: Warwick edinburgh mental well-being scale
WHO: World Health Organisation
